# X-Ray-Induced Damage to the Submandibular Salivary Glands in Mice: An Analysis of Strain-Specific Responses

**DOI:** 10.1089/biores.2015.0017

**Published:** 2015-06-01

**Authors:** Mana Kamiya, Tomoyuki Kawase, Kazuhide Hayama, Makoto Tsuchimochi, Kazuhiro Okuda, Hiromasa Yoshie

**Affiliations:** ^1^ Division of Oral Bioengineering, Department of Tissue Regeneration and Reconstitution, Institute of Medicine and Dentistry, Niigata University, Niigata, Japan.; ^2^ Division of Periodontology, Department of Oral Biological Science, Institute of Medicine and Dentistry, Niigata University, Niigata, Japan.; ^3^ Advanced Research Center, The Nippon Dental University School of Life Dentistry at Niigata, Niigata, Japan.; ^4^ Department of Oral and Maxillofacial Radiology, The Nippon Dental University School of Life Dentistry at Niigata, Niigata, Japan.

**Keywords:** apoptosis, inflammation, saliva, submandibular gland, T lymphocytes, X-ray

## Abstract

Radiation therapy for head and neck cancers often causes xerostomia (dry mouth) by acutely damaging the salivary glands through the induction of severe acute inflammation. By contrast, the mechanism underlying the X-ray-induced delayed salivary dysfunction is unknown and has attracted increasing attention. To identify and develop a mouse model that distinguishes the delayed from the acute effects, we examined three different mouse strains (C57BL/6, ICR, and ICR-nu/nu) that showed distinct T-cell activities to comparatively analyze their responses to X-ray irradiation. Three strains were irradiated with X-rays (25 Gy), and functional changes of the submandibular glands were examined by determining pilocarpine-induced saliva secretion. Structural changes were evaluated using histopathological and immunohistochemical examinations of CD3, cleaved poly (ADP-ribose) polymerase (PARP), and Bcl-xL. In C57BL/6 mice, the X-ray irradiation induced acute inflammation accompanied by severe inflammatory cell infiltration at 4 days postirradiation, causing substantial destruction and significant dysfunction at 2 weeks. Fibrotic repair was observed at 16 weeks. In ICR-nu/nu mice, the inflammation and organ destruction were much milder than in the other mice strains, but increased apoptotic cells and a significant reduction in salivary secretion were observed at 4 and 8 weeks and beyond, respectively. These results suggest that in C57BL/6 mice, X-ray-induced functional and structural damage to the salivary glands is caused mainly by acute inflammation. By contrast, although neither acute inflammation nor organ destruction was observed in ICR-nu/nu mice, apoptotic cell death preceded the dysfunction in salivary secretion in the later phase. These data suggest that the X-ray-irradiated ICR-nu/nu mouse may be a useful animal model for developing more specific therapeutic methods for the delayed dysfunction of salivary glands.

## Introduction

Dry mouth, also called xerostomiais, is often caused by radiation therapy in head and neck cancer patients.^1–3^ X-ray irradiation atrophies salivary glands, attenuating their function and consequently reducing saliva secretion.^4–6^ Detailed histopathological examinations have demonstrated that X-ray irradiation induces acute inflammation by stimulating inflammatory cell infiltration, resulting in chronic inflammation in which damaged and atrophied salivary glands are repaired with fibrous tissue.^4–7^ It is generally thought that X-ray irradiation predominantly damages the salivary glands indirectly through acute inflammation.^4,8^ However, it has also been reported that X-ray irradiation causes cytoplasmic vacuole formation and nuclear condensation.^4,9,10^ These changes in cellular components appear distinct from the indirect damage mediated by acute inflammation. Although the mechanisms underlying these changes remain unclear, it is likely that X-ray irradiation damages the salivary glands not only by inflammation-dependent pathways but also by other direct pathways that have not yet been identified.

It has generally been accepted that cellular X-ray sensitivity is dependent on cell division.^11^ According to this concept, less differentiated highly proliferative cells, such as malignant cells, are highly sensitive to X-rays. Radiation therapy is therefore used to eliminate cancer cells. However, while the cells of the salivary glands are highly differentiated and lowly proliferative, patients receiving low-dose radiation therapy suffer from dry mouth for many years, even after treatment has ended,^12–15^ demonstrating that the salivary glands are also highly radiosensitive.^16–19^

To better understand the pathology of X-ray-induced dry mouth, it is important to distinguish acute responses from organ damage and to generate an animal model that accurately reflects the potential direct action of X-ray irradiation on the salivary cells. In a preliminary study, we screened the radiosensitivities of submandibular glands in various mouse strains and found that C57BL/6 and nude mice differed. C57BL6 mice exhibit a dominant Th1 cell activity,^20,21^ whereas ICR-nu/nu mice genetically lack thymi and, consequently, lack functional T cells.^22^ In this study, we therefore used these two mouse strains as well as ICR, a parent strain of the nude mice, and compared their responses to X-ray irradiation by focusing on the structure and function of the salivary glands.

## Materials and Methods

### Mouse strains

Five-week-old female mice (C57BL/6, ICR, ICR-nu/nu) were obtained from Charles River Laboratories Japan, Inc. (Yokohama, Japan) and housed for at least 1 week before experimentation.

The care and use of the mice complied with the Guiding Principles for the Care and Use of Animals and was approved by the Niigata University and the Nippon Dental University School of Life Dentistry at Niigata.

### X-ray irradiation of the submandibular glands

Mice were fixed in a dorsal position on wooden stages and anesthetized with isoflurane (Wako Pure Chemicals, Osaka, Japan) using an inhalation anesthesia system for small experimental animals (DS Pharma, Osaka, Japan). The field of irradiation encompassing the submandibular salivary glands was adjusted to a 10-mm slit. The mice were then irradiated with 25 Gy using a linear accelerator (PRIMUS M2-6300; Siemens Medical Solutions USA, Inc., Malvern, PA). The control animals were fixed and anesthetized, but not irradiated.

### Evaluation of the salivary secretion rate

At 2, 4, 8, and 16 weeks postirradiation, the volume of secreted saliva was determined as described below. Mice were injected peritoneally with pilocarpine hydrochloride (2 mg/kg; Sigma-Aldrich, St. Louis, MO), and after 5 min, the pilocarpine-stimulated saliva was collected for 10 min and weighed using an electric balance (Mettler-Toledo, Tokyo, Japan). The salivary secretion rate was quantified by dividing the salivary weight by the time and the body weight.

### Determination of survival rate and body weight

The mice were continuously observed and weighed every 7 days for up to 5 weeks. The survival rate was determined by dividing the number of surviving mice by the total number of irradiated mice.

### Macroscopic observation of body surface

To observe changes in body surface, mice were fixed on stages and photographed. In the nonirradiated control mice, the hair around the target regions was shaved to visualize the skin condition. To detect typical signs of inflammation, such as swelling and heat, the irradiated regions were further examined by palpation.

### Histopathological and immunohistochemical examinations

After euthanasia by intraperitoneal injection of pentobarbital (Somnopentyl^®^; Kyoritsu Seiyaku, Tokyo, Japan), the submandibular salivary glands were excised and fixed overnight in 10% neutralized formalin at 4°C. The organs were dehydrated, embedded in paraffin, and sectioned for histopathological and immunohistochemical examination. Briefly, for histopathological examinations, the resulting sections were subjected to hematoxylin–eosin (HE) or Masson's trichrome staining.^23^ All dye solutions were obtained from Muto Pure Chemicals Co., Ltd. (Tokyo, Japan), and Masson's trichrome staining was performed according to the manufacturer's instructions.

For immunohistochemical staining, the sections were treated for 30 min with 0.3% H_2_O_2_ in methanol to block the endogenous peroxidase activity followed by L.A.B Solution (Polysciences, Inc., Warrington, PA) for 5 min at ambient temperature to retrieve antigen.^23^ After blocking with Block-Ace (DS Pharma), the sections were treated overnight at 4°C with the following primary antibodies: mouse monoclonal anti-CD3 (1:100 in dilution; Santa Cruz Biotechnology, Santa Cruz, CA); rabbit monoclonal anti-cleaved PARP (1:50 in dilution; Abcam, Cambridge, MA); or rabbit monoclonal anti-Bcl-xL (1:500; Abcam). After three washes with phosphate-buffered saline containing 0.1% Tween-20, the sections were treated with horseradish peroxidase-conjugated anti-mouse or anti-rabbit IgG (1:100, diluted with IMMUNO SHOT Mild [CosmoBIO, Tokyo, Japan]) (Life Technologies, Carlsbad, CA) for 60 min at ambient temperature and visualized with diaminobenzidine (DAB; Kirkegaard & Perry Laboratories, Inc., Gaithersburg, MD). The counterstain was performed using hematoxylin.

The number of positive cells was determined by image analysis using WinROOF software (Mitani Corporation, Fukui, Japan).

### Statistical analyses

The data are reported as the mean value±standard deviation. Statistical analyses were performed to compare the mean values using Student's *t*-test (SigmaPlot 12.5; Systat Software, Inc., San Jose, CA). *p*-Values <0.05 were considered statistically significant.

## Results

### The effects of X-ray irradiation on body weight, survival, and salivary secretion

In a preliminary study, we investigated the relationship between the irradiation dose and survival rate and found that, as reported elsewhere,^17,24,25^ X-ray irradiation at 15 Gy or lower did not significantly reduce the survival rate of C57BL/6 mice. However, to clearly contrast nude mice from other mouse strains, we used a relatively higher dose in this study.

**FIG. 1.**
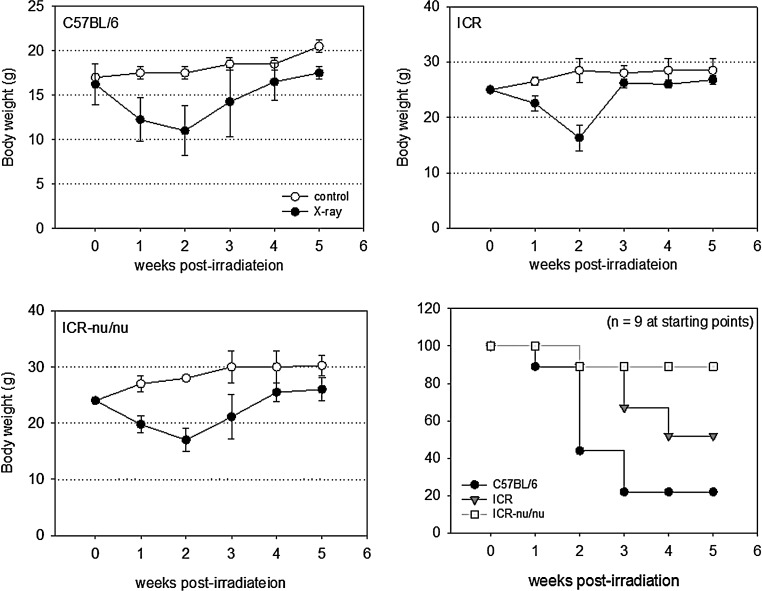
Time course of changes in body weights and survival rates of X-ray-irradiated mice (25 Gy). Nine animals from each mouse strain were used at the starting points.

In this study, X-ray irradiation (25 Gy) induced similar biphasic changes in body weight in all the mouse strains tested (Fig. 1). For up to 2 weeks postirradiation, body weights decreased with time by 35–40% (vs. control) and subsequently reverted to increasing with time. However, the survival rate varied by the mouse strain; C57BL/6 and ICR-nu/nu mice demonstrated the lowest and highest survival rates, respectively.

**FIG. 2.**
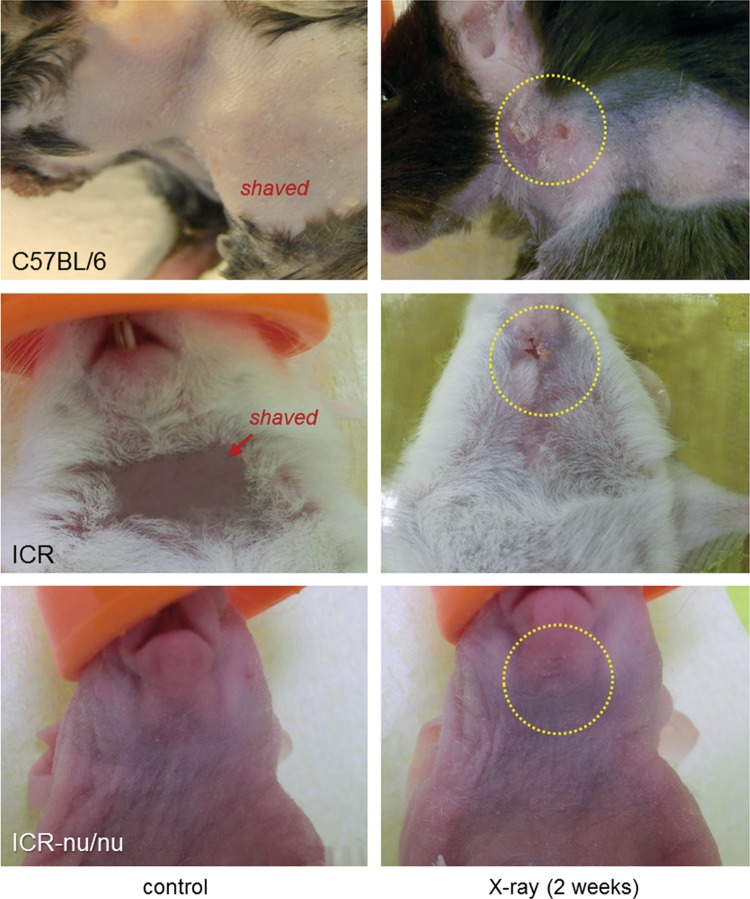
Skin ulcerations in the irradiated fields at 2 weeks postirradiation (25 Gy). To observe skin condition, hair was shaved in control mice. Similar observations were obtained with three additional mice from each strain.

Macroscopic examination at 2 weeks postirradiation demonstrated an appreciable reddening of the skin, hair loss, and ulceration in the irradiated fields in both the C57BL/6 and ICR mice (Fig. 2); however, these changes were more obvious in the C57BL/6 mice. Similar ulceration was clearly observed in the mucosa of the pharynx and larynx of the irradiation fields in the C57BL/6 mice (data not shown). By contrast, in the ICR-nu/nu mice, neither similar changes nor typical signs of inflammation such as swelling and heat were detected by macroscopic examination and palpation.

**FIG. 3.**
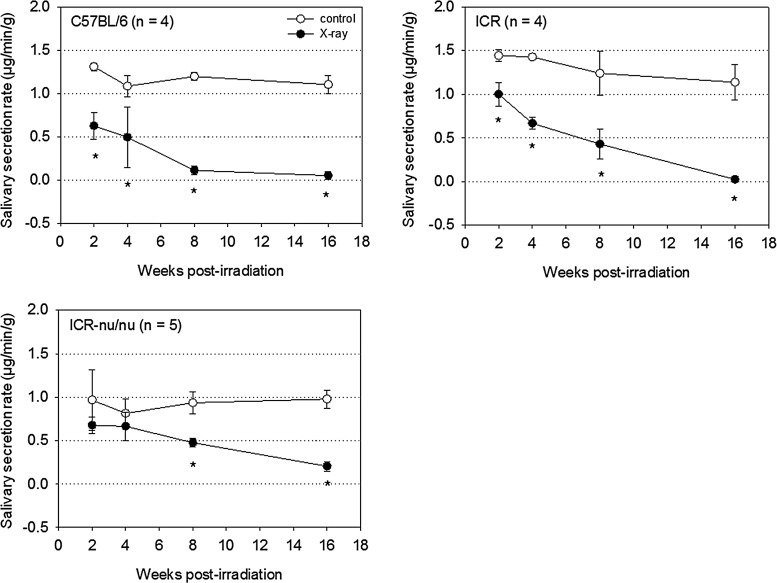
Time course of the changes in salivary secretions in X-ray-irradiated mice (25 Gy). *n*=4 or 5. *p<0.05 compared with each corresponding control.

Salivary secretion was evaluated from 2 to 16 weeks postirradiation. X-ray irradiation significantly reduced the salivary secretion rate in both C57BL/6 and ICR mice as early as at 2 weeks (Fig. 3). By contrast, the salivary secretion was significantly reduced after 8 weeks in ICR-nu/nu mice.

### The effects of X-ray irradiation on the morphology and inflammation of the submandibular salivary glands

**FIG. 4.**
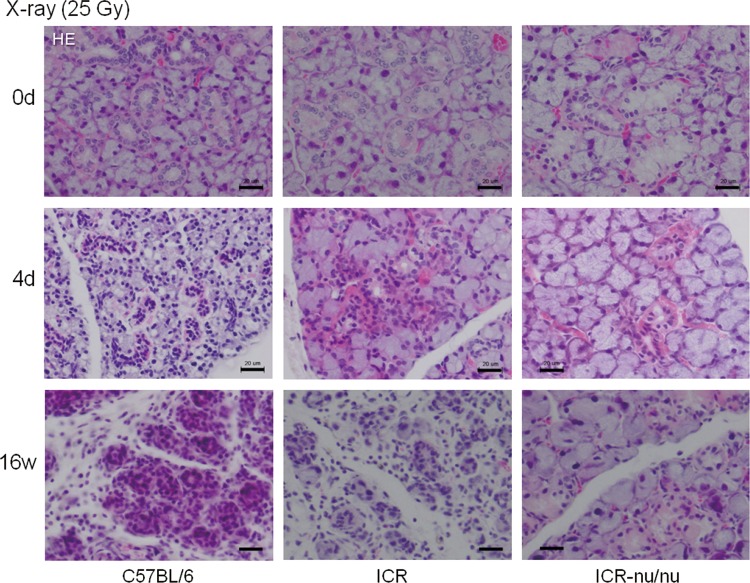
Time course of morphological changes in the submandibular salivary glands in X-ray-irradiated mice (25 Gy). The salivary glands were retrieved at 4 days and 16 weeks postirradiation, and the paraffin sections were subjected to hematoxylin–eosin staining. Similar observations were obtained from two additional mice from each strain. Bar=20 μm.

X-ray-induced acute and chronic inflammation was examined histologically using HE staining (Fig. 4). At day 4 in both C57BL/6 and ICR mice, X-ray irradiation caused acute inflammation with an increased infiltration of inflammatory cells, primarily T lymphocytes. Although inflammatory cells were abundant in C57BL/6 mice, they were observed in limited numbers around the intercalated ducts in ICR mice. These infiltrating cells were still present after the structural destruction of salivary glands at 16 weeks, indicating a shift to chronic inflammation in these mice. By contrast, no appreciable inflammatory response was observed in ICR-nu/nu mice, and the normal structure of salivary glands was preserved at 16 weeks.

**FIG. 5.**
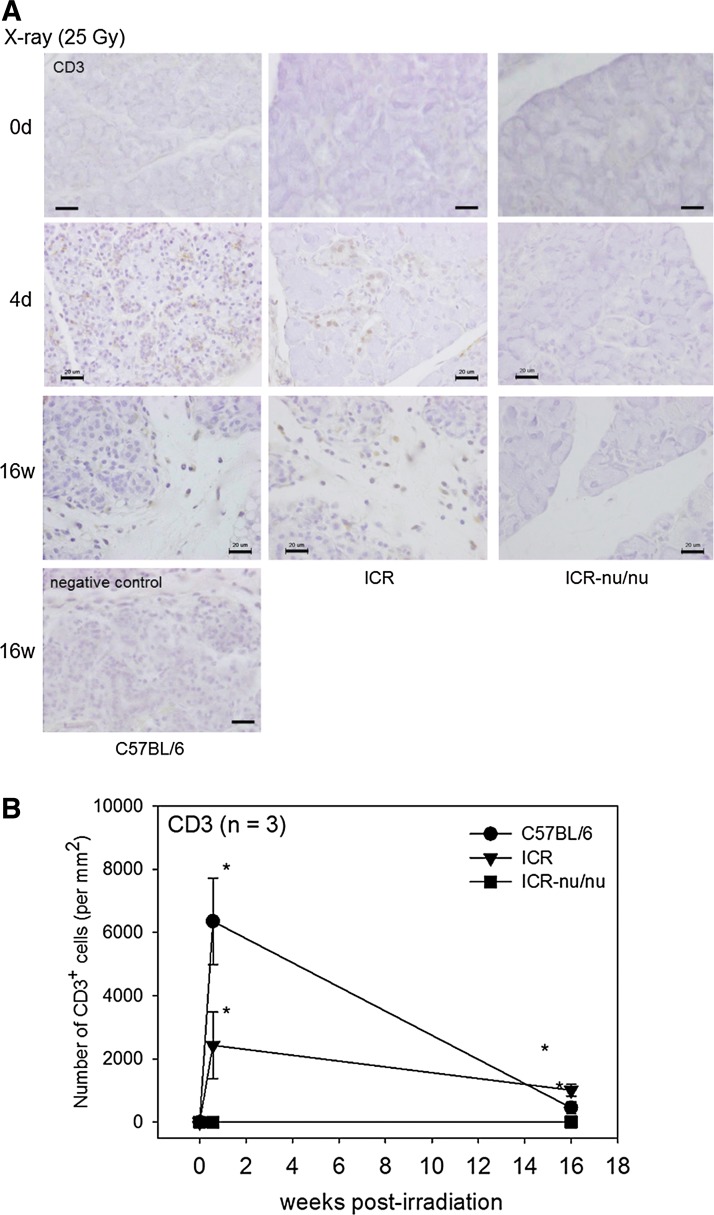
The effects of X-ray irradiation on the infiltration of CD3^+^ T cells and the construction of the submandibular salivary glands. **(A)** Immunohistochemical data of X-ray-irradiated mice (25 Gy). The salivary glands were retrieved at 4 days and 16 weeks postirradiation, and the paraffin sections were subjected to immunohistochemical staining. The negative control is shown at the bottom left of the figure. Bar=20 μm. **(B)** Time course of the changes in CD3^+^ T-cell infiltration in the submandibular salivary glands. These immunohistochemical data were quantified and plotted in a graph. *n*=3. *p<0.05 compared with each result obtained at 0 day (nonirradiated control).

The X-ray-induced infiltration of CD3^+^ T cells was examined immunohistochemically using an anti-CD3 antibody (Fig. 5). C57BL/6 mice are known to exhibit the dominant Th1 cell activity,^20,21^ whereas ICR-nu/nu mice genetically lack thymi and, consequently, functional T cells.^22^ It was therefore predicted that CD3^+^ T cells would infiltrate during acute inflammation in C57BL/6, but not in ICR-nu/nu mice. In C57BL/6 mice, the number of CD3^+^ T cells dramatically increased immediately after X-ray irradiation, followed by a rapid decrease to near basal levels within 16 weeks. In ICR mice, the number of CD3^+^ T cells that infiltrated during acute inflammation did not substantially decrease to basal levels within 16 weeks, but were sustained in chronic inflammation. In contrast, no significant infiltration of CD3^+^ T cells was observed in ICR-nu/nu mice. The immunohistochemical data were quantitated and plotted (Fig. 5, bottom). At day 4, the extent of CD3^+^ T-cell infiltration was C57BL/6 >> ICR>ICR-nu/nu, and at 16 weeks, it was ICR>C57BL/6>ICR-nu/nu. These data are consistent with the HE-staining data.

**FIG. 6.**
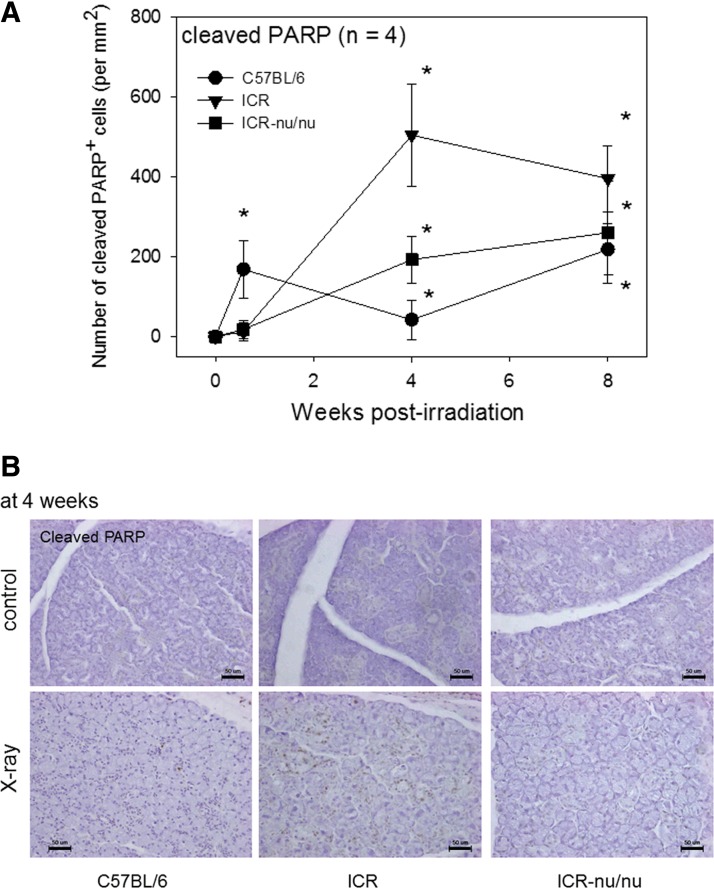
The effects of X-ray irradiation on cleaved PARP^+^ apoptotic cell death in the submandibular salivary glands. **(A)** Time course of the changes in cleaved PARP^+^ cell appearance in X-ray-irradiated mice (25 Gy). The salivary glands were retrieved at 4 days, 4 and 8 weeks postirradiation, and the paraffin sections were subjected to immunohistochemical staining. These immunohistochemical data were quantified and plotted. *n*=4. *p<0.05 compared with each corresponding (nonirradiated) control, which was constantly sustained below the detection limit. **(B)** Immunohistochemical data of the cleaved PARP^+^ cells at 4 weeks. Scale bar=50 μm.

**FIG. 7.**
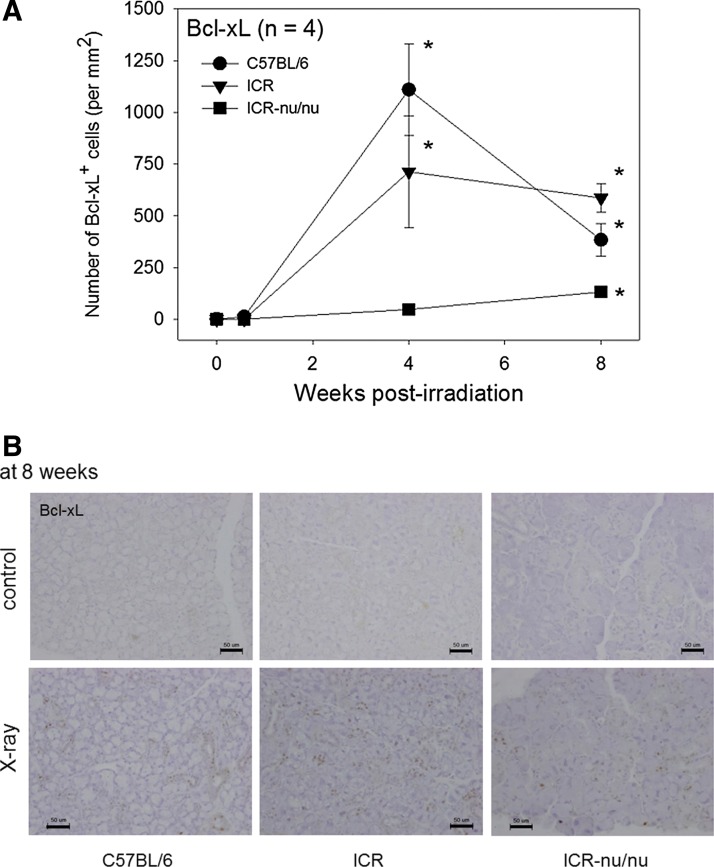
The effects of X-ray irradiation on Bcl-xL^+^ cells resisting apoptotic cell death in the submandibular salivary glands. **(A)** Time course of changes in Bcl-xL^+^ cell appearance in X-ray-irradiated mice (25 Gy). The salivary glands were retrieved at 4 days, 4 and 8 weeks postirradiation, and the paraffin sections were subjected to immunohistochemical staining. These immunohistochemical data were quantified and plotted. *n*=4. *p<0.05 compared with each corresponding (nonirradiated) control, which was constantly sustained below the detection limit. **(B)** Immunohistochemical data of Bcl-xL^+^ cells at 8 weeks. Scale bar=50 μm.

The X-ray-induced appearance of cleaved PARP^+^ and Bcl-xL^+^ cells was examined immunohistochemically (Figs. 6 and 7). Cleaved PARP and Bcl-xL are known as apoptotic and antiapoptotic markers, respectively.^26–29^ The immunohistochemical data were quantitated and plotted (Fig. 6, top panel). In C57BL/6 mice, X-ray irradiation immediately increased cleaved PARP^+^ cells at 4 days, while significant increases in cleaved PARP^+^ cells were observed at 4 weeks in ICR and ICR-nu/nu mice. The initial changes varied by the mouse strain, whereas by 8 weeks, the level of cleaved PARP^+^ cells was similar across mouse strains. Immunohistochemical data at 4 weeks are shown in Figure 6 (bottom panels). Significant age-related increases in cleaved PARP^+^ cells were not observed in the control mice for all the strains tested at this time point.

On the other hand, in both the C57BL/6 and ICR mice, X-ray irradiation increased Bcl-xL^+^ cells to maximum levels at 4 weeks (Fig. 7, top panel). Thereafter, the Bcl-xL^+^ cells decreased in C57BL/6 mice, whereas these cells did not significantly decrease with the shift to chronic inflammation in ICR mice at 8 weeks. Despite the lack of an appreciable inflammatory response in X-ray-irradiated ICR-nu/nu mice, Bcl-xL^+^ cells were detected, peaking at 8 weeks, which was later than in other mouse strains. Immunohistochemical data at 8 weeks are shown in Figure 7 (Bottom panels). Significant age-related increases in Bcl-xL^+^ cells were not observed in any of the control mouse strains tested at this time point.

**FIG. 8.**
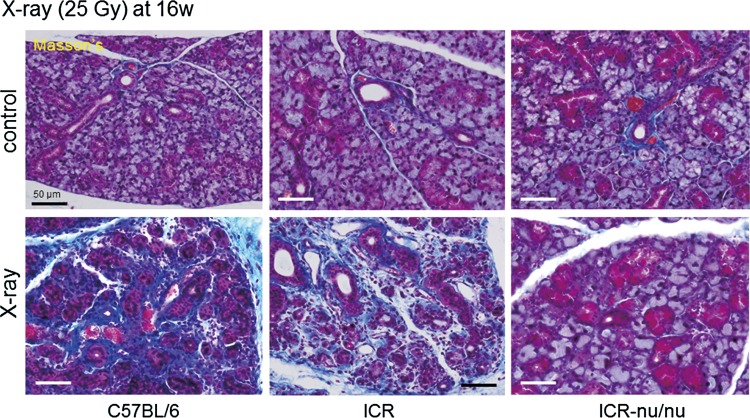
The effects of X-ray irradiation on collagen deposition in the submandibular salivary glands of X-ray-irradiated mice (25 Gy). The salivary glands were retrieved at 16 weeks postirradiation, and the paraffin sections were subjected to Masson's trichrome staining. The blue staining in the extracellular matrix represents collagen. Bar=50 μm.

Factors influencing the regression of acute inflammation and the shift to chronic inflammation on repair/regeneration of the submandibular salivary glands were examined histochemically (Fig. 8). In C57BL/6 mice, in which the most severe acute inflammation was observed, submandibular salivary glands were widely and largely replaced with newly formed, collagen-enriched fibrotic tissue at 16 weeks. In ICR mice, a similar, although less substantial, fibrous replacement was observed. By contrast, in ICR-nu/nu mice, significant collagen deposition was not induced at this time point.

## Discussion

Based on data obtained from animal experiments and clinical investigations, X-ray-induced dysfunction of the salivary glands is generally thought to be mediated predominantly by acute inflammation.^4,8^ To our knowledge, however, no reliable reports have demonstrated the mechanism of X-ray action on the salivary glands at a cellular and molecular level. To investigate the involvement of inflammation in the effects of X-rays, we used three different mouse strains that showed distinct T-cell activities to compare responses to X-ray irradiation using histological and immunohistochemical methods.

Consistent with T-cell activities reported elsewhere,^20–22^ these three animal models showed different inflammatory responses to X-ray irradiation. Macroscopic examination revealed ulcers in the skin, the larynx, and the pharynx within the irradiation fields with the severity of the ulceration in descending order as follows: C57BL/6>ICR >> ICR-nu/nu. The same trend was observed for the survival rate. In addition to these basic changes, the onset of salivary gland dysfunction was different in each mouse strain. In C57BL/6 and ICR mice, salivary secretion was significantly suppressed at the early phases postirradiation (∼2 weeks), whereas in ICR-nu/nu mice, the suppression of salivary secretion was observed at 8 weeks and beyond.

Histological and immunohistochemical examination revealed morphological and cellular changes that led to macroscopic and functional changes. In response to X-ray irradiation, the salivary glands were acutely infiltrated by primarily CD3^+^ T lymphocytes, and the submandibular salivary glands of C57/BL6 mice were the most severely destroyed. At 16 weeks, the majority of the damaged salivary glands had been replaced with fibrotic tissue. By contrast, X-ray irradiation did not cause increased inflammation and fibrotic tissue repair in the ICR-nu/nu mice. Interestingly, despite the absence of inflammation, there were significant increases in cells expressing apoptotic and antiapoptotic markers at 4 and 8 weeks postirradiation, respectively.

Consistent with the literature,^4,8^ the present findings not only suggest that X-ray irradiation destroys the structure of the submandibular salivary glands by inducing severe acute inflammation but also indicate that X-ray irradiation may lead to salivary gland dysfunction without causing inflammation. These two mechanisms are distinguishable by their onset; the former is an acute response, whereas the latter is a delayed response. The characteristics of these responses are further summarized in Table 1. In the acute response, X-ray irradiation activates the immune system to induce acute inflammation. Depending on the T-cell activity, the structure of the salivary gland is either moderately or extensively destroyed, after which the destroyed organs are repaired with fibrotic tissue, likely through a T-cell-mediated transforming growth factor-β-dependent mechanism.^30–33^ By contrast, in the delayed response, X-ray irradiation stimulates salivary gland cells to induce apoptotic cell death. The peak number of apoptotic cells occurred at 4 weeks postirradiation, which preceded the onset of salivary gland dysfunction in ICR-nu/nu mice. Therefore, although the apoptotic cells are not yet identified in this study, it seems likely that the delayed dysfunction is due to a lack of regeneration by stem/progenitor cells.^8,34–37^

**Table 1.** **Two Distinct Pathways Proposed to Mediate the X-Ray-Induced Dysfunction of the Submandibular Salivary Glands**

	Acute response	Delayed response
Primary target	Immune system	Salivary stem and/or progenitor cells
Primary response	Acute inflammation	Not clear
Initial changes	Structural destruction at organic levels	Targeted apoptotic cell death
Onset of dysfunction	Early phase	Late phase
Secondary response	Repair with fibrotic tissue	Not clear

In head and neck cancer patients receiving radiation therapy, it has been speculated that, as observed in the C57BL/6 mice, a combination of both responses leads to the onset of dry mouth syndrome. According to Konings et al.,^38^ the onset of salivary gland dysfunction can be classified into four groups: acute (0–10 days), early (10–60 days), intermediate (60–120 days), and late (120–240 days). Although the mechanism for the onset of each stage is unknown, several studies have suggested that X-ray irradiation directly damages the mesenchymal stem cells and/or progenitor cells in intercalated ducts to induce a delayed onset of dysfunction.^18,34–39^ Although X-ray irradiation does not induce severe acute inflammation in ICR-nu/nu mice, it generated a significant number of apoptotic cells at 4 weeks postirradiation and significantly reduced saliva secretion at 8 weeks. Based on the time course of functional and cellular changes, we suggest that the X-ray-irradiated ICR-nu/nu mouse reflects the delayed intermediate onset of salivary gland dysfunction observed in humans. Therefore, the X-ray-irradiated ICR-nu/nu mouse may be a useful model for developing more specific therapeutic methods for the intermediate or late types of X-ray-induced dry mouth.

During strategic treatment planning, it is important to thoroughly understand the mechanism of disease onset. To efficiently regenerate X-ray-damaged salivary glands, it is necessary to develop and properly use specific therapeutic methods for the treatment of acute and delayed dysfunction. Without the transplantation of mesenchymal stem cells and/or salivary gland progenitor cells or the recruitment of bone marrow stem cells, the regeneration of salivary glands to cure dry mouth seems unlikely, particularly in the case of delayed dysfunction.^40–42^

## Summary

We prepared X-ray-irradiated animal models from three mouse strains with different T-cell activities and performed pathological analysis. C57BL/6 mice responded to X-ray irradiation with severe acute inflammation that immediately and severely destroyed the structure of the submandibular salivary glands. In ICR-nu/nu mice, there was no appreciable inflammatory response to X-ray irradiation. However, a significant increase in apoptotic cells preceded salivary dysfunction at 4–8 weeks. Therefore, we propose that the X-ray-irradiated ICR-nu/nu mouse is a useful animal model for studying X-ray-induced dry mouth, especially for developing more specific therapeutic methods for the delayed dysfunction of salivary glands.
